# Assessment of the Laser Beam Welding of Galvanized Car Body Steel with an Additional Organic Protective Layer

**DOI:** 10.3390/ma16020670

**Published:** 2023-01-10

**Authors:** Jacek Górka, Wojciech Suder, Monika Kciuk, Sebastian Stano

**Affiliations:** 1 Department of Welding Engineering, Silesian University of Technology, Konarskiego Str. 18a, 44-100 Gliwice, Poland; 2 Welding and Additive Manufacture, Cranfield University, Cranfield MK43 0AL, UK; 3 Department of Engineering Materials and Biomaterials, Silesian University of Technology, Konarskiego Str. 18a, 44-100 Gliwice, Poland; 4 Łukasiewicz Research Network—Institute of Welding, Bl. Czesława 16-18, 44-100 Gliwice, Poland

**Keywords:** car body sheets, laser beam welding, linear welding energy, mechanical properties, corrosion resistance

## Abstract

This study discusses the effect of laser beam welding parameters on the structure, mechanical properties and corrosion resistance of 1.2 mm thick galvanized sheets made of low-carbon steel DC04 provided with a ZE36/36 GardoprotectOC2BU organic coating. The test laser beam butt welded joints were made without the filler metal, using a variable welding rate, where linear welding energy was restricted within the range of 30 J/mm to 90 J/mm. The joints were subjected to non-destructive tests, destructive tests and corrosion resistance tests. The tests revealed the possibility of making joints meeting the criteria specified in the ISO 15614-11 standard. Regardless of the value of linear welding energy applied in the process, all the joints were characterised by high mechanical and plastic properties. It was noticed that an increase in linear welding energy from 30 J/mm to 90 J/mm was accompanied by the widening of the weld and that of the heat-affected zone (HAZ). In addition, an increase in linear welding energy was accompanied by a decrease in the maximum weld hardness to approximately 250 HV0.2. In the HAZ, hardness was restricted within the range of 190 HV0.2 to 230 HV0.2 and decreased along with increasing linear welding energy. In the static tensile test, regardless of the value of linear welding energy, the test specimen ruptured in the base material. In the bend test, regardless of the value of linear welding energy, a bend angle of 180° was obtained without partial tear or scratches; unit elongation was restricted within the range of 29% to 42%. The electrochemical tests and experiments performed in the salt spray chamber revealed the very high effectiveness of the corrosion protections against aggressive chloride ions.

## 1. Introduction

In the automotive industry, steel is responsible for most of the car weight. Figures for the year 2017 show that steel and cast elements constitute approximately 55% of the car weight, whereas plastics are responsible for approximately 11% and aluminium alloys constitute approximately 9% of the vehicle weight [1,2,3,4,5,6,7]. The development of the automotive industry entails an increased demand for new structural materials. Competing carmakers try to reach the largest market share and, because of this, try to satisfy consumers’ expectations related to increasingly good product quality. In addition to satisfying customers’ needs, high quality positively affects users’ safety and increases the level of vehicle reliability [8,9,10,11,12,13,14,15]. When manufacturing high-quality components, particular attention should be paid to the quality of welded joints. Until recently, the most common technology used when joining car body elements has been resistance spot welding. However, because of its numerous advantages, laser beam welding is increasingly often applied in the aforesaid part of the manufacturing process. The selection of the best joining method for state-of-the-art engineering and car body production is a delicate matter. Taking into account the fact that the design engineer has, basically, only three parameters that must be combined in order to obtain an optimum solution (i.e., geometrical shape, material and joining method), the selection of an appropriate joining technique is of great importance. The foregoing partly explains why new assembly technologies are enjoying growing popularity at the cost of traditional welding methods [16,17,18,19,20,21,22,23,24,25].

Galvanized steel sheets are used in various industrial sectors as zinc coatings provide the former with favourable corrosion resistance in adverse weather conditions. The welding of zinc-coated materials requires complying with special HSE regulations as the joining process may trigger the emission of harmful zinc oxide (zinc melting point being 419.5 °C) [26,27,28,29,30,31]. In addition, molten zinc can penetrate steel grain boundaries, ultimately reducing the plasticity of joints. During intense heating, evaporating zinc may lead to weld porosity. The stability of optimum welding process parameters and the quality of edge preparation are key factors in laser beam welding. An increase in the welding rate narrows the width of the weld face as well as that of the heat-affected zone and of the weld root. Exceeding a certain welding rate value may lead to the formation of welding imperfections, such as the excessively large reinforcement on the root or the incompletely filled groove [13,32,33,34,35,36].

Galvanized sheets provided with organic coatings, designed having in view high anti-corrosion properties combined with press-formability and weldability, are enjoying an increasingly high popularity. During the manufacturing process, metal sheets are first provided with a zin coating and, afterwards, with a thin (up to approximately 20 μm) layer based on epoxy resin with an addition of metal particles (up to between 65% and 75% of the volume). The aforesaid material was invented as early as in the 1970’s, where the high content of metallic compounds in the coating layer was used to facilitate arc welding processes [37,38,39,40]. The market offer includes first and second-generation anti-corrosive coatings. The technologies used to obtain the first-generation coatings are applied during the chromium-free pre-treatment of sheets, where the coating thickness is restricted within the range of 2.5 μm to 4.0 μm. Corrosion protection obtained in such a process is between three and four times higher than that obtainable in the conventional galvanising process without the application of the prime coat. In turn, the second-generation prime coatings are applied during the chromium-free pre-treatment of sheets, which were previously subjected to electro or hot galvanizing processes. The coating thickness restricted within the range of 3 μm to 5 μm provides corrosion protection being between 6 and 8 times higher than that obtained during galvanizing without additional painting and two-fold higher than that obtainable by means of the first-generation prime coatings. The laser beam welding of advanced steels is also possible during the fabrication of sheets provided with protective coatings. In such cases, the making of joints is identical to the process used during the welding of mild steel. However, it should be noted that the welding of steels provided with zinc coatings necessitates leaving a narrow gap (between 0.1 mm and 0.2 mm) between elements to be joined (to prevent the formation of porosity). Interestingly, it is also possible to weld such sheets without leaving the gap, yet in such a case, the joining process requires the application of two laser beams, where the first beam heats up the zinc layer (causing the evaporation of the latter), whereas the second beam is used to weld the elements [41,42,43,44,45].

In the conducted own research, an attempt was made to assess the influence of the linear energy of laser beam welding on the properties and corrosion resistance of butt-welded joints made of galvanized steel with an organic coating applied. For such thin sheets used in the automobile industry, they are usually lap welded, the article presents the results of butt welding because they are interesting from the research point of view; there are few works on joining such thin sheets in the automotive industry [46,47].

## 2. Materials and Methods

The research work discussed in the article aimed to identify the effect of laser beam welding parameters on the structure, mechanical properties and corrosion resistance of 1.2 mm thick galvanized sheets made of steel DC04 provided with a ZE36/36 GardoprotectOC2BU protective coating. The test sheets made of low-carbon, cold workable and deep-drawing steel DC04 were provided with a zinc coating and an organic layer. The zinc and organic coatings protect the sheet surface against the corrosive activity of an external environment. The steel protected in the above-presented manner is used in the production of car bodies. The chemical composition and properties of the test material are presented in Table 1 and Table 2.

### 2.1. Welding Process

**Figure 1:**
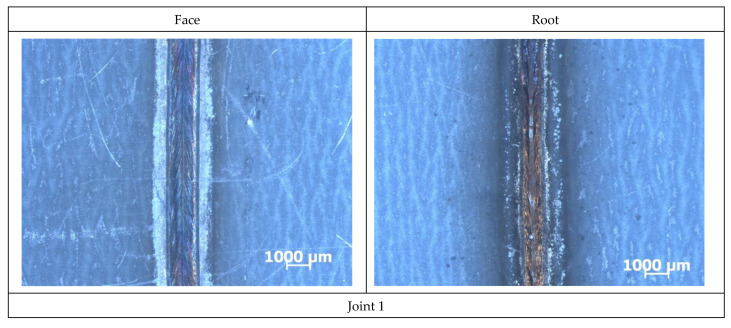

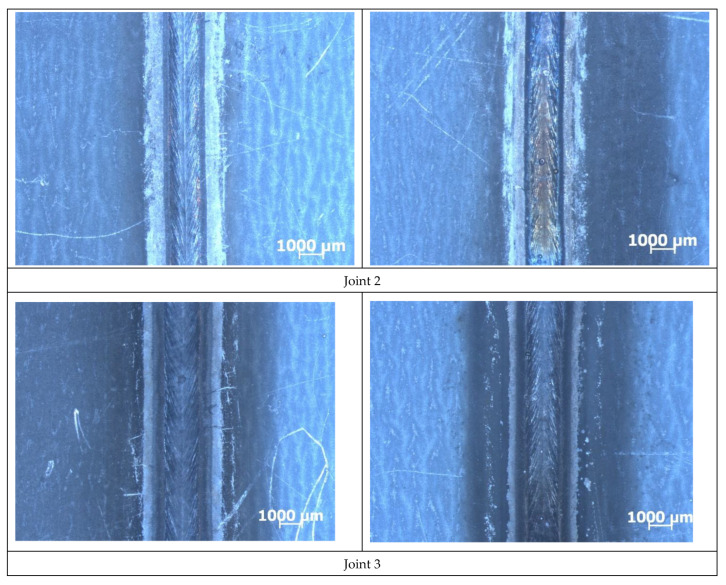
View of welded joints from the weld face and weld root side, welding parameters and joint markings in accordance with the data in Table 3.

The sheets made of galvanized steel DC04 provided with the organic coating had dimensions of 300 mm × 150 mm × 1.2 mm. The test pieces were subjected to cleaning and degreasing. The butt test joints, made using a TruLaser Robot 5120 robotic station equipped with a TruDisk 12002 disk laser, were welded without the filler metal. The welding process was carried out using a CFO head equipped with a fiber optic cable with a diameter of 400 μm. The focal length of the collimator lens was 200 mm, the focal length was 300 mm, the focal diameter of the beam was 0.6 mm. The specimens were made using various welding rates. The welding process parameters are presented in Table 3. The weld face and the weld root are presented in Figure 1.

### 2.2. Tests of Welded Joints

First, the welded joints were subjected to non-destructive tests: visual tests, magnetic-particle tests and radiographic tests. Visual tests (VT) were carried out based on requirements of the PN-EN ISO 17637:2011 standard. After visual tests, in order to detect possible cracks, surface tests were carried out using the magnetic particle method based on the guidelines of the standards PN-EN ISO 3059:2005, PN-EN ISO 9934-2:2003, PN-EN ISO 9934-3:2003. MR 72 agent was used as a white contrasting base, while the magnetic powder MR 76S was used to carry out the tests, the tests were carried out with the use of a yoke electromagnet. In order to reveal volumetric defects, radiographic tests were carried out based on the PN-EN 1435 standard with a CERAM type 235 X-ray tube with a beam of X-rays with a diameter of d = 2 mm at voltage U = 180 kV, the intensity I = 3 mA, using reinforcing covers OW −0.15 mm. The test results were recorded on AGFA type C5 photographic film, using the exposure time t = 2.3 min and the focal length f = 700 mm. The 13FEEN bar indicator was used to assess image quality.

After the non-destructive tests, macro and microscopic metallographic tests were carried out to determine structural changes. Macroscopic metallographic tests performed using an Olympus SZX9 stereoscopic light microscope; the specimens were subjected to etching in Adler’s reagent. Microscopic metallographic tests performed using a NIKON ECLIPSE MA100 light microscope; the specimen were subjected to etching in Nital. The strength properties were determined on the basis of a static tensile test and a bending test with stretching from the side of the weld face and from the side of the weld root. The hardness measurement was carried out using the Vickers method along one measuring line. A corrosion potential test was carried out performed using a three-electrode measurement system and an ATLAS 0531 EU potentiostat-galvanostat (Atlas-Sollich, Rebiechowo, Poland). The electrolyte used in the tests was the aqueous solution of sodium chloride having a mass concentration of 3%; the reference electrode was a saturated silver-silver chloride electrode (Ag, AgCl), whereas the counter electrode was made of stainless steel. The recording of polarisation curves followed the determination of corrosion potential (after approximately one hour); measurements were performed at a potential change rate of 0.01 V/s. corrosion tests performed in accordance with the requirements specified in the PN-EN ISO 9227 standard, using a CC450iP salt spray chamber (Ascott, Singapore). The specimens were exposed to the 3.5% salt mist of the aqueous solution of sodium chloride at a temperature of 35 °C and a humidity of 100%; the time of exposure amounted to 96 h. In order to assess corrosion changes, tests were carried out—fractographic tests, using a ZEISS SteREO Discovery V12 stereoscopic microscope at 100× magnification.

## 3. Results and Discussion

The visual and magnetic particle tests of the joints made using a variable welding rate restricted within the range of 30 J/mm to 90 J/mm did not reveal the presence of surface-breaking welding imperfections such as cracks, porosity, incomplete fusion, lacks of penetration, burn-through, etc.

**Figure 2:**
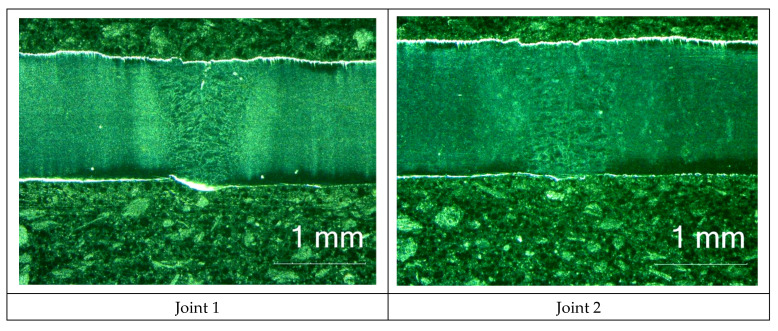

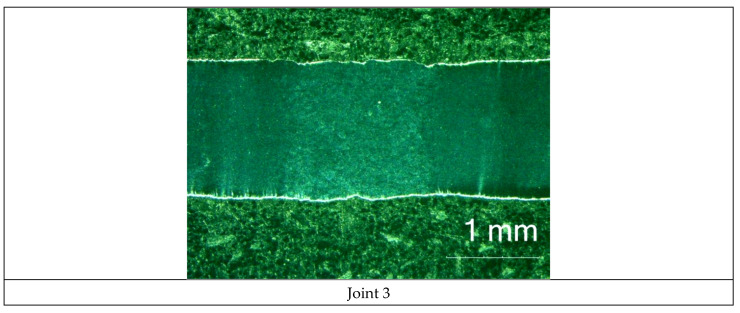
Macrostructure of welded joints.

All of the welded joints represented quality level B in accordance with the ISO 13919-1 standard. The macroscopic tests did not reveal the presence of welding imperfections in the weld area or in the HAZ (Figure 2). The surface of the test sheets was provided with the protective layer.

**Figure 3:**
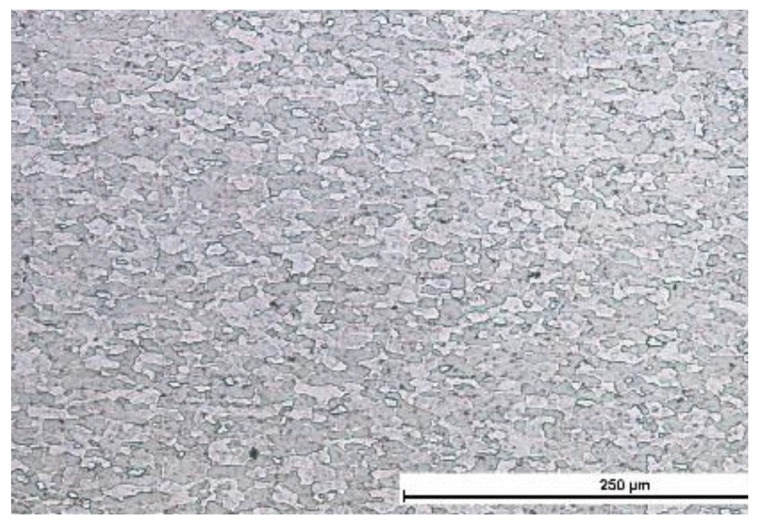
Microstructure of base material.

**Figure 4:**
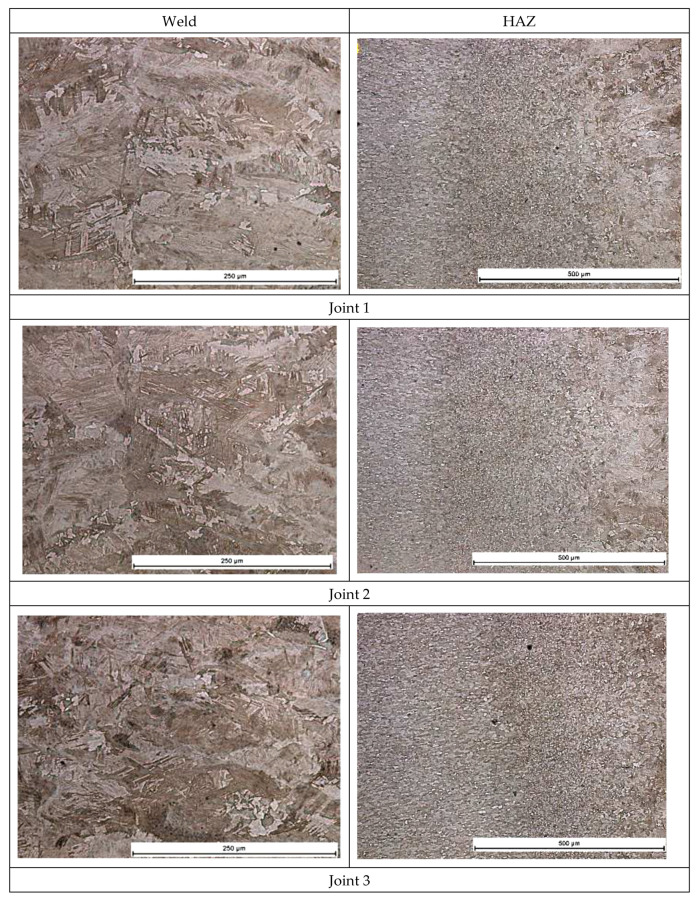
Microstructure of joints made with variable linear welding energy.

The microscopic metallographic tests revealed the effect of linear welding energy on the structure and the grain size in the weld area and in the HAZ. The base material is characterized by a ferritic-pearlitic structure, Figure 3. In cases of the welds made using the lowest linear welding energy (i.e., 30 J/mm) it was possible to observe both the ferritic-bainitic structure and martensitic islands. An increase in linear welding energy was accompanied by an increase in a ferrite content and a decrease in a martensite content in the weld. The structure of the weld in the joint made using a linear welding energy of 90 J/mm was ferritic-bainitic. The HAZ was characterised by grain size variability. In the HAZ area, an increase in linear welding energy was accompanied by a decrease in the grain size (see Figure 4).

**Figure 5:**
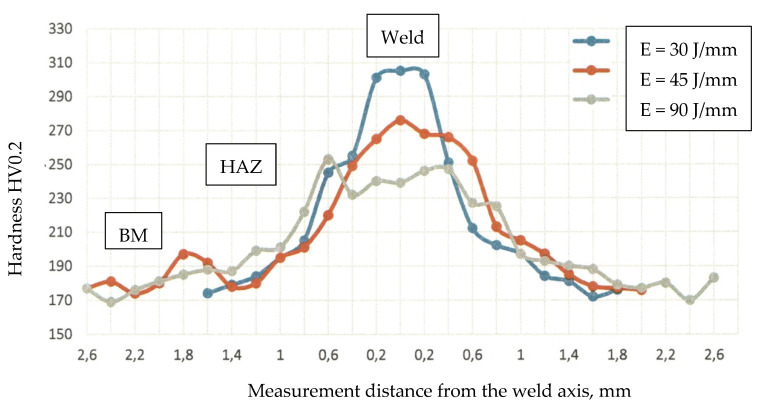
Hardness distribution of welded joints.

The analysis of the hardness test results confirmed the observations of the microscopic metallographic tests. The hardness test results (Figure 5) depicted an increase in hardness in the HAZ and in the weld if compared with that of the base material (amounting to approximately 175 HV0.2). The highest hardness value was obtained in the weld of joint 1, made using the lowest linear welding energy (i.e., 30 J/mm) and characterised by the presence of martensitic structures. An increase in linear welding energy was accompanied by a decrease in the maximum hardness of the weld to approximately 250 HV0.2. In the HAZ area, hardness values were restricted within the range of 190 HV0.2 to 230 HV0.2 and decreased along with increasing linear welding energy (which was connected with the grain size—see Figure 5).

**Figure 6:**
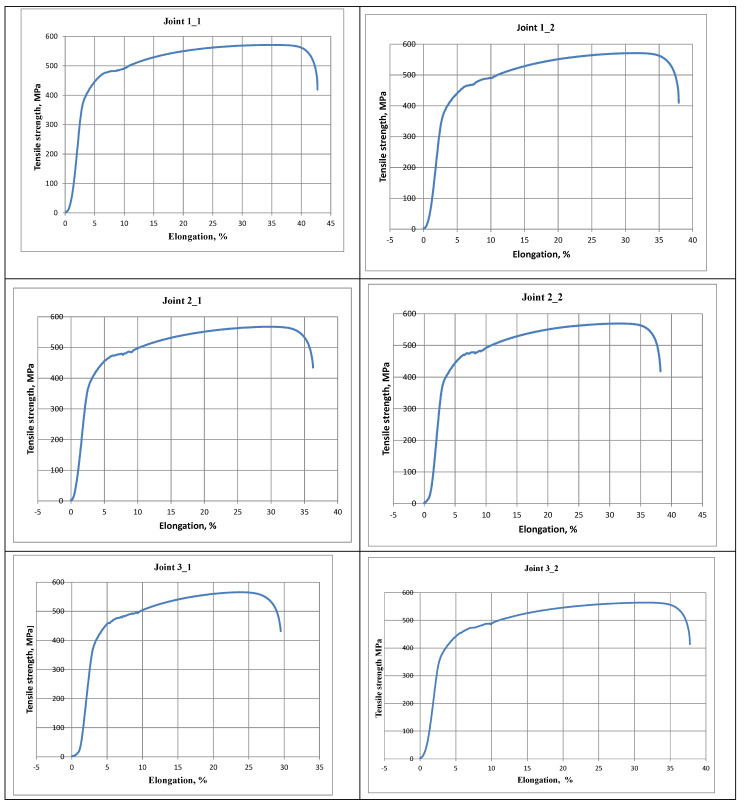
Tensile strength chart.

The static tensile tests revealed that all of the joints were characterised by high tensile strength (of approximately 570 MPa) and that the rupture, regardless of linear welding energy, took place outside the welded joint area (see Table 4, Figure 6).

Both the face and root bend tests of the butt weld enabled the obtainment of a bend angle of 180°, which indicated the high plastic properties of the test joint.

**Figure 7:**
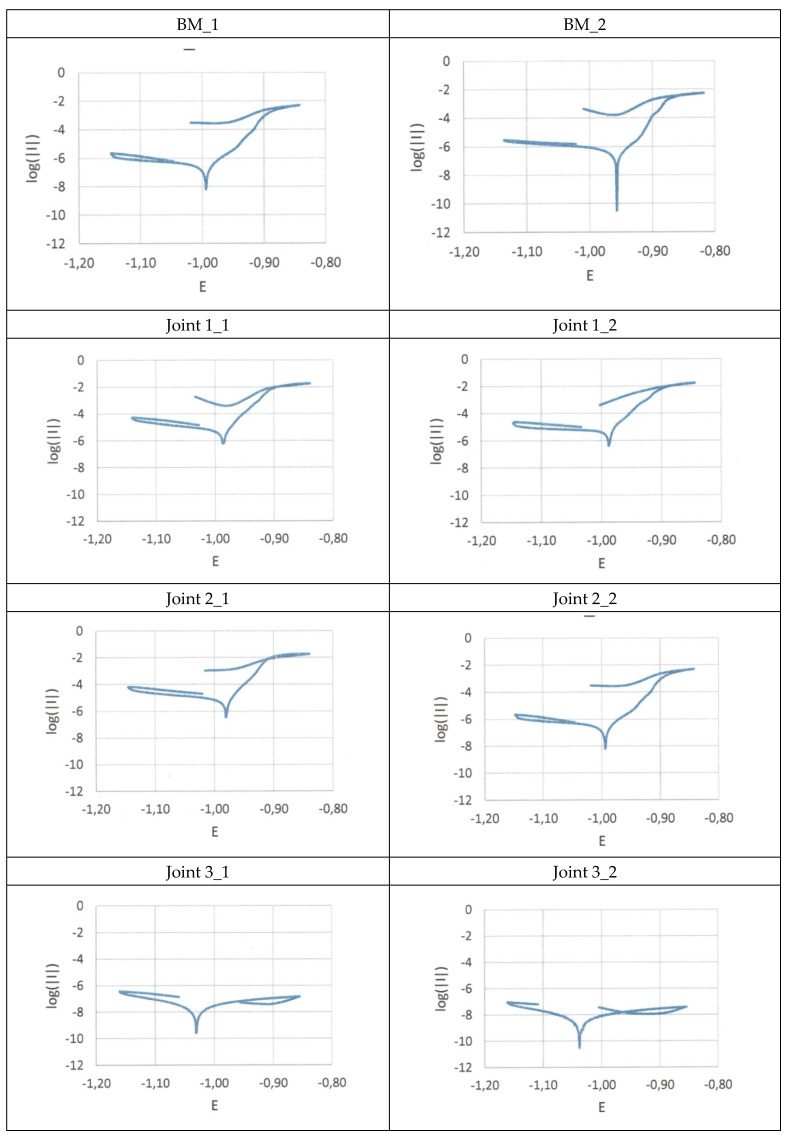
Polarization curves.

The electrochemical tests aimed to determine the corrosion resistance of the test material and the test joints under predefined corrosive conditions. The corrosion tests were performed in a 3.5% NaCl environment, simulating seawater. The recording of polarisation curves followed the determination of corrosion potential (after approximately one hour, see Figure 7). Measurements were performed at a potential change rate of 0.01 V/s. The corrosion potential measurement results are presented in Table 5.

The characteristics of the corrosion resistance of the initial material and of the welded joints were determined using linear polarisation, aimed to identify the kinetic parameters of the corrosion process, i.e., current density (I_cor_), corrosion potential (E_cor_) and polarisation resistance (R_p_). Corrosion potential E_cor_ of the base material was restricted within the range of −0.95 V to −0.99 V, the values of polarisation resistance were restricted within the range of 51.8 kΩ cm^2^ to 67.6 kΩ cm^2^, whereas corrosion potential amounted to −0.99 V and −0.95 V respectively.

The analysis of the parameters determined in the tests revealed that the highest corrosion resistance was that of welded joints nos. 3_1 and 3_2, which was manifested by the lowest values of current density (amounting to 4.60250 × 10^−9^ and 1.96567 × 10^−9^ V) as well as by the highest values of polarisation resistance (amounting to 1.46 cm^2^ and 2.88 MΩ cm^2^ respectively). The service life and reliability of the organic coatings on the zinc layer surface (the so-called metal-organic coatings) were primarily affected by the initial state of the zinc coating, the manner of surface preparation and the proper methodology of coating deposition. In addition, the deposition of the coatings on the galvanized material was impeded by the lack of sufficient adhesion of the coating to the zinc surface, which resulted, among other things, from the amphoteric nature of zinc, its chemical activity and the reactivity of zinc corrosion products. The organic coating provides a continuous barrier protecting the steel surface against aggressive ions present in a corrosive environment. In the event of the perforation of the organic coating, the protective function of the steel surface is taken over by the zinc coating. Zinc, as metal characterised by lower electrochemical potential than that of iron, becomes the anode in the corrosion cell and undergoes destruction, thus protecting the steel substrate.

**Figure 8:**
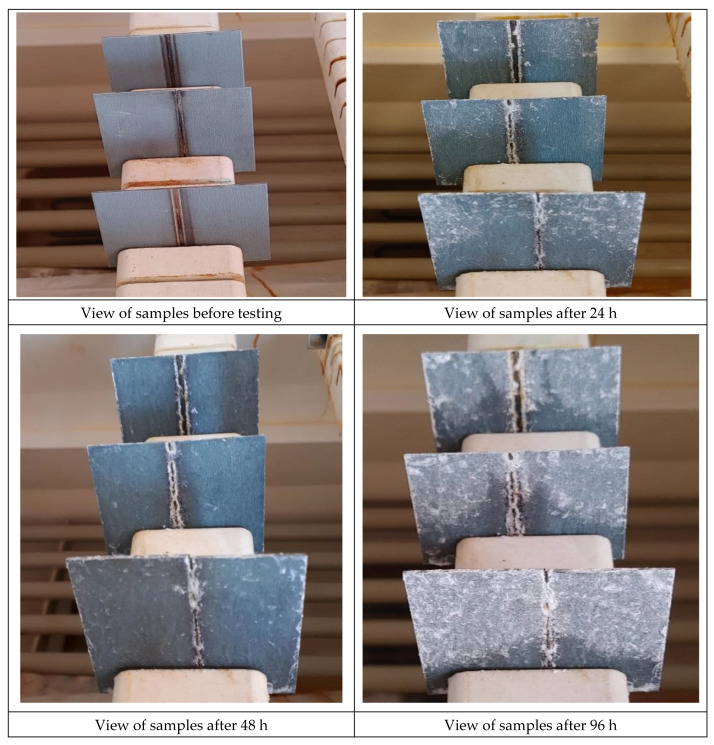
View of the samples during the salt spray test.

The reduced corrosion resistance of welded joints nos. 1_1, 1_2, 2_1 and 2_2 was probably connected with the loss or damage to a part of the zinc galvanic coating triggered by the welding process (Table 5). The defective coating surface enabled corrosion agents to penetrate the gaps in the surface, which led to the development of the local corrosion of the steel substrate. The corrosion resistance tests were also performed in a salt spray chamber, in accordance with the requirements specified in the PN-EN ISO 9227 standard. The specimens were placed in the salt spray chamber and exposed to the 5% salt mist of the aqueous solution of sodium chloride at a temperature of 35 °C (Figure 8). The time of exposure amounted to 96 h.

Corrosion-induced changes of the test specimens were assessed in a gravimetric manner, on the basis of their mass depletion and the depletion of mass in relation to the surface (Table 6). The mass depletion of the specimens placed in the salt spray chamber was slight and, after 96 h’ exposure, amounted to a maximum of 4.6552 g/m^2^. In accordance with the PN EN 9227 standard, concerning galvanized specimens, the value of mass depletion below 50 ± 25 g/m^2^ after 48 h is satisfactory.

**Figure 9:**
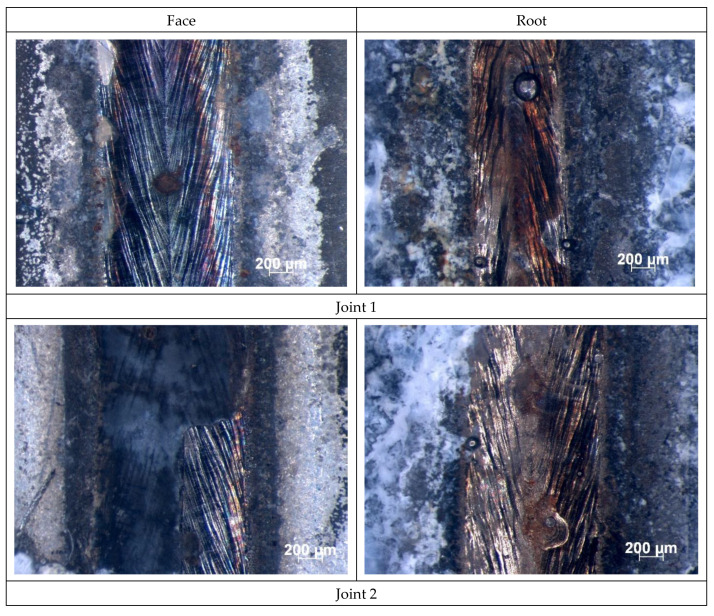

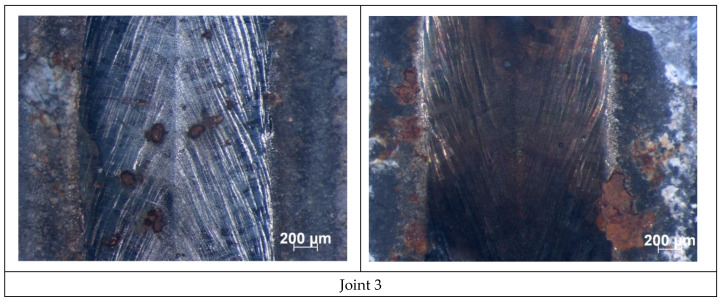
Surface of welded joints after corrosion tests in 5% NaCl solution.

The identification of the type and nature of corrosion-induced damage required the performance of tests concerned with the morphology of the test specimen surface after the corrosion tests. The morphology-related tests were performed using a SteREO Discovery V12 stereoscopic microscope (ZEISS). The methodology applied in the tests enabled the proper assessment of the service life of corrosion protections. The surface of the specimens subjected to analysis contained few corrosion centres located both within the weld and in the heat-affected zone (Figure 9).

The areas affected by corrosion-triggered damage were characterised by varied geometry, yet, because of the slight depletion of specimen mass, corrosion pit penetration was probably not significantly deep. The areas affected by corrosion were those where the corrosion protection was damaged or eliminated entirely through welding. The local defects were characteristic of pitting corrosion (taking place in an autocatalytic manner) and probably resulted from the penetration of aggressive chloride ions (Cl^−^) deep inside the unprotected material. The base material, where anti-corrosion layers were not damaged, was not affected by the corrosion environment.

## 4. Conclusions

The tests concerning 1.2 mm thick laser beam welded joints made of low-carbon galvanized steel DC04 provided with the ZE36/36 GardoprotectOC2BU organic coating revealed the possibility of obtaining joints satisfying the requirements of the ISO 15614-11 standard. Regardless of linear welding energy, all of the joints were characterised by favourable mechanical and plastic properties.

The analysis of macrostructural cross-sectional images made it possible to determine the effect of present welding parameters on the geometrical dimensions of the test welds. The above-named analysis revealed that an increase in linear welding energy from 30 J/mm to 90 J/mm led to the widening of the weld and that of the HAZ. The microscopic tests revealed the fine-grained structure of the base material and the grain growth within the weld area.

The microscopic test results confirmed those obtained in the hardness measurements. The highest hardness was obtained in the weld of joint 1 (made using a linear welding energy of 30 J/mm), which was connected with the presence of martensitic structures. An increase in linear welding energy was accompanied by a decrease in the maximum hardness of the weld to approximately 250 HV0.2. In the HAZ areas, hardness values were restricted within the range of 190 HV0.2 to 230 HV0.2 and decreased along with increasing linear welding energy, which, in turn, was related to the grain size. In the static tensile test, regardless of linear welding energy, the specimen ruptured in the base material, which confirmed high mechanical properties of the test joints.

The tensile strength of the test specimens was restricted within the range of 564 MPa to 571 MPa. It was possible to observe a slight decrease in R_m_ along with an increase in linear welding energy. In the bend test, regardless of the value of linear welding energy, a bend angle of 180° was obtained without partial tear or scratches. The values of unit elongation were restricted within the range of 29% to 42%, thus confirming the high plastic properties of the joints.

The electrochemical tests and experiments performed in the salt spray chamber revealed the very high effectiveness of the corrosion protections against aggressive chloride ions. The values of corrosion current density and those of corrosion potential made it possible to identify correlation log ǀI_cor_ǀ = f(E_cor_). In each case it was possible to observe the characteristic shape of polarisation curves.

The analysis of the parameters identified in the tests and of all the characteristics revealed that the highest corrosion resistance was observed in the welded joints designated as 3_1 and 3_2. The foregoing was manifested by the low value of corrosion current and the high value of polarisation resistance. Slight damage observed in the weld area and in the HAZ resulted from the welding process and the subsequent heat-triggered removal of the protective layers.

## Figures and Tables

**Table 1:** Chemical composition of the DC04 steel.

Element Concentration, wt %			
C	P	S	Mn
≤0.08	0.03	0.03	0.40

**Table 2:** Selected properties of DC04 steel in the tempered state.

Yield Strength Re, MPa	Tensile Strength Rm, MPa	Elongation A80, %	Hardness, HV
Min. 460	490–590	37	155–185

**Table 3:** Parameters of the welding process of test joints.

Joint Designation	Beam Power, W	Welding Speed V, m/min	Welding Line Energy, J/mm
Joint 1	1500	3	30
Joint 2	1500	2	45
Joint 3	1500	1	90

**Table 4:** Static tensile test results and sample dimensions.

Joint Designation	Tensile Strength R_m_, Mpa	Elongation A_t_, %	Sample Thickness a_0_, mm	Sample Width b_0_, mm	S_0_, mm^2^	The Place of the Breakup
Joint 1_1	571	42	1.2	10	12	BM
Joint 1_2	571	37	1.2	10	12	BM
Joint 2_1	567	36	1.2	10	12	BM
Joint 2_2	569	38	1.2	10	12	BM
Joint 3_1	565	29	1.2	10	12	BM
Joint 3_2	564	37	1.2	10	12	BM

**Table 5:** Corrosion potential measurement results.

Joint Designation	Current Density, I_cor_, A/cm^2^	Corrosion Potential, E_cor_, V	Polarisation Resistance, R_pol_, Ωcm^2^
BM_1	5.88212 × 10^−8^	−0.993562625	67,636.270
BM_2	3.83675 × 10^−8^	−0.956986276	51,785.238
Joint 1_1	1.34020 × 10^−6^	−0.986044568	2667.442
Joint 1_2	1.16184 × 10^−6^	−0.986414818	2390.899
Joint 2_1	1.51086 × 10^−6^	−0.978703074	1957.343
Joint 2_2	1,19116 × 10^−6^	−0.987951811	2634.453
Joint 3_1	4.60250 × 10^−9^	−1.029928000	1,459,048.000
Joint 3_2	1.96567 × 10^−9^	−1.020356000	2,877,017.000

**Table 6:** Results of mass loss and mass loss relative to the surface after corrosion tests.

	Joint 1	Joint 2	Joint 3
Dimensions, mm	60 × 67	52 × 67	52 × 67
Weight before test, g	39.4752	34.2620	34.1426
Weight after test, g	39.4696	34.2458	34.1264
Loss of weight, g	0.0056	0.0162	0.0162

## Data Availability

The data presented in this study are available on request from the corresponding author. The data are not publicly available because the authors do not wish to publish Supplementary Materials.
